# The complete chloroplast genome of *Madhuca hainanensis* (Sapotaceae), an endemic and endangered timber species in Hainan Island, China

**DOI:** 10.1080/23802359.2021.1878954

**Published:** 2021-03-15

**Authors:** Yong Wang, Hai-Tao Wang, Yu-Kai Chen, Jing Yu, Yong Yang

**Affiliations:** a Ministry of Education Key Laboratory for Ecology of Tropical Islands, College of Life Sciences, Hainan Normal University, Haikou, China; b Institute of Cotton, Hebei Academy of Agriculture and Forestry Sciences, Shijiazhuang, China; c College of Horticulture, Hainan University, Haikou, China

**Keywords:** *Madhuca hainanensis*, chloroplast genome, Illumina sequencing, phylogenetic analysis

## Abstract

*Madhuca hainanensis* Chun & F.C.How is an endangered and endemic species in Hainan Island, and it was ranked as a VU (Vulnerable) species in China. In this study, we reported its complete chloroplast (cp) genome sequence based on high throughput sequencing data. The complete cp genome was 159,630 bp in length, containing two short inverted repeat (IRs) of 26,093 bp, a large single copy (LSC) region of 88,846 bp and a small single copy (SSC) region of 18,598 bp. Totally, the cp genome contained 131 genes, including 86 protein coding genes (PCG), eight rRNA genes and 37 tRNAs. The GC contents of this genome was 36.8%. A maximum likelihood (ML) phylogenetic analysis indicates that *M. hainanensis* is closely related to *Sinosideroxylon wightianum*.

The genus Madhuca in family Sapotaceae is widely distributed in South and Southeast Asia, including 85 species in the world. Among these, 2 species (*Madhuca hainanensis* and *Madhuca pasquieri*) are only distributed in Southern China, and *Madhuca hainanensis* Chun & F.C.How (https://www.ipni.org/n/787485-1) is endemic species in Hainan Island, China (Francisco-Ortega et al. 2010; Dai et al. 2013; Lin et al. 2018). It is extremely narrow distribution, occuring only in tropical, montane rain forests and evergreen forest at high altitudes of about 1000 m in Hainnan Island (Li and Huang 1996; Zhang and Ma 2008). In China, *M. hainanensis*, a valuable tropical timber species, is always used for shipbuilding, axles, sports equipment, mechanical appliance, furniture and bridges, because it has excellent characteristics such as dark reddish brown in appearance, dense in structure, tough in texture, and resistant to rot (Chen 1990; Huang et al. 2011). In addition, its seed is rich in oil, up to 55%, which can be used for food and soap making, and its bark contains rich tannin (Huang et al. 2011). However, *M. hainanensis* natural resources have been depleted due to long term use and unreasonable harvesting (Mo et al. 2007; Dai et al. 2013). At present, *M. hainanensis* was ranked as a VU (Vulnerable) species in China (World Conservation Monitoring Centre 1998), and only few researches related to its communitties, seedling conservation and human impacts on genetic diversity (Dai et al. 2013; Zhai et al. 2015). In this study, we herein assembled the complete chloroplast genome of *M. hainanensis* (GeneBank: MT909828) based on sequences data obtained with the Illumina HiSeq platform. Its cp genome sequence would contribute fundamental information to further phylogenetical and protective studies of this plant.

The sample of *M. hainanensis* was collected from Bawangling national nature reserve (N19°05′, E109°10′), Hainan Island, China, and deposited at the botany laboratory of Hainan Normal University, Haikou, China (Sample accession number: HS-1068). Total DNA was extracted from fresh leaves using the modified CTAB method (Doyle 1987).The genome was sequenced on an Illumina Novaseq 6000 platform (Illumina, San Diego, CA, USA) with 150 bp paired-end reads. In total, approximately 4.88 Gb of clean reads data with an average coverage of 516× were obtained after quality filtering and trimming. The cp genome were assembled by SPAdes v.3.11.0 software (Bankevich et al. 2012) with cp genome of *Lucuma. nervosa* (MH018545) as reference. The assembled cp genome genes were then annotated using PGA (Qu et al. 2019), coupled with manual check and adjustment.

The complete cpDNA of *M. hainanensis* was 159,630 bp in length. The genome was a typical quadripartite structure, containing a pair of short inverted repeat (IRs) of 26,093 bp, which was separated by a large single copy (LSC) region of 88,846 bp and a small single copy (SSC) region of 18,598 bp. The complete cp genome contained 131 genes, including 86 protein coding genes (PCG), 8 rRNA genes and 37 tRNAs. Among these genes, 15 genes (*trnK-UUU*, *rps16*, *trnG-UCC*, *atpF*, *rpoC1*, *trnL-UAA*, *trnV-UAC*, *petB*, *petD*, *rpl16*, *rpl2*, *ndhB*, *trnI-GAU*, *trnA-UGC* and *ndhA*) harbored a single intron and two genes (*clpP*, *ycf3*) had two introns. The majority of genes in *M. hainanensis* occurred as a single copy. The overall GC content of *M. hainanensis* cp genome is 36.8%.

To clarify the phylogenetic of *M. hainanensis*, a maximum likelihood (ML) phylogenetic tree was reconstructed by MAGA7 software using concatenated protein sequences of 64 single copy genes which were found in all 14 chloroplast genomes of Ebenales and *Magnolia pyramidata* (*Magnoliaceae*) as an out-group (Figure 1). The result showed that *M. hainanensis* is closely related to *Sinosideroxylon wightianum*, and all representatives of Ebenales were clustered into one monophyletic clade. Our result will provide useful information for genetic evolution and convervation genetics of this endangered plant.

**Figure 1.**
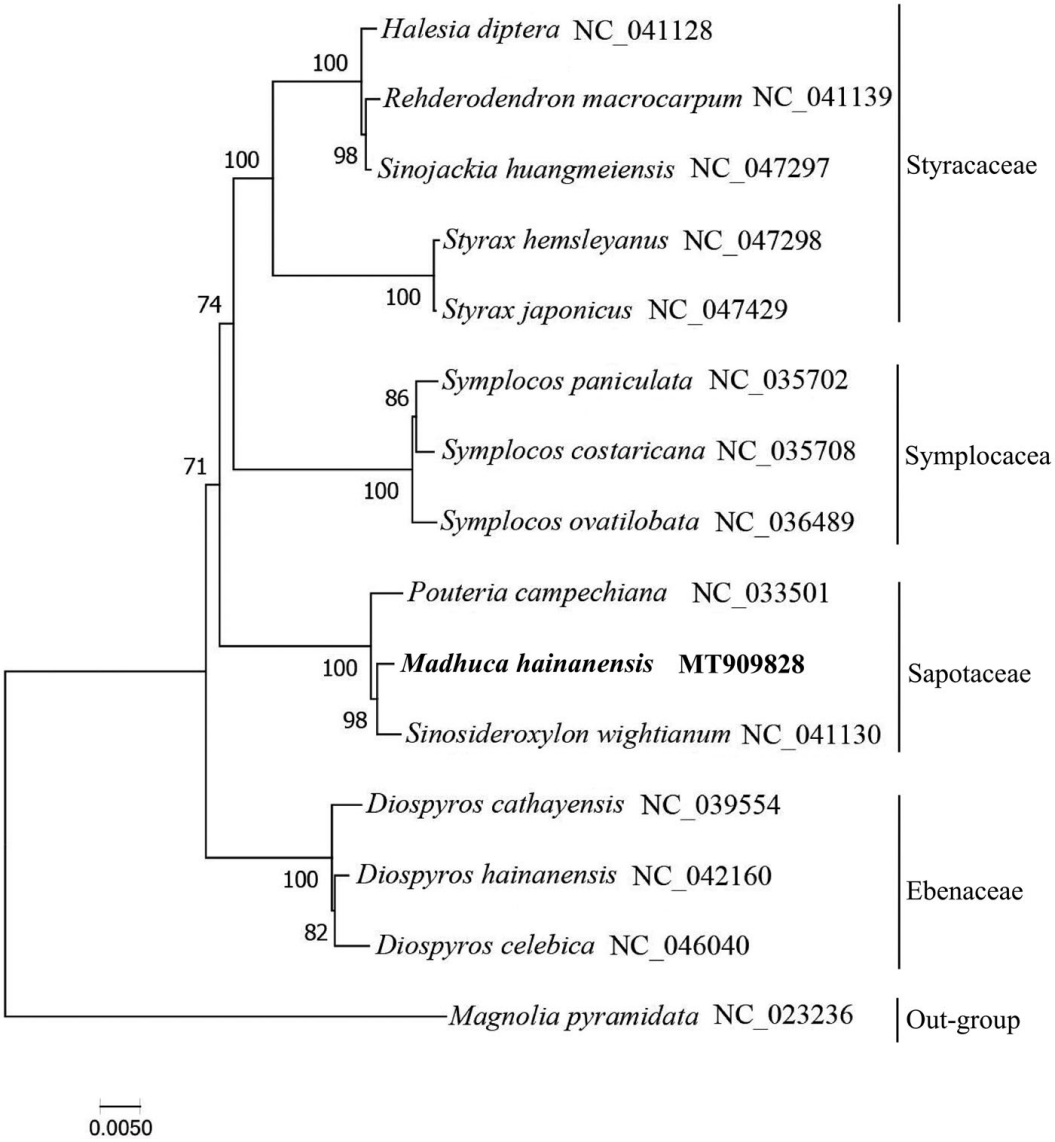
Maximum likelihood tree of *M. hainanensis* based on concatenated protein sequences of 64 single copy genes found in all 15 chloroplast genome. Numbers in the nodes were bootstrap values from 1000 replicates.

## Data Availability

The genome sequence data that support the findings of this study are openly available in GenBank of NCBI at https://www.ncbi.nlm.nih.gov/ under the accession number MT909828. The associated BioProject, SRA and Bio-Sample numbers are PRJNA668681, SRP287064, and SAMN16418375 respectively.
